# The Effect of Rosemary (*Rosmarinus officinalis*) and Blackcurrant Extracts (*Ribes nigrum*) Supplementation on Performance Indices and Oxidative Stability of Chicken Broiler Meat

**DOI:** 10.3390/ani11041155

**Published:** 2021-04-17

**Authors:** Kamil Sierżant, Małgorzata Korzeniowska, Janusz Orda, Aneta Wojdyło, Florence Gondret, Tomasz Półbrat

**Affiliations:** 1 Department of Animal Nutrition and Feed Quality, Wrocław University of Environmental and Life Sciences, 51630 Wrocław, Poland; janusz.orda@upwr.edu.pl (J.O.); tomasz.polbrat3@gmail.com (T.P.); 2 Department of Animal Products Technology and Quality Management, Wrocław University of Environmental and Life Sciences, 51630 Wrocław, Poland; malgorzata.korzeniowska@upwr.edu.pl; 3 Department of Fruit, Vegetable and Plant Nutraceutical Technology, Wrołcaw University of Environmental and Life Sciences, 51630 Wrocław, Poland; aneta.wojdylo@upwr.edu.pl; 4 PEGASE INRAE, Institut Agro, 35590 Saint-Gilles, France; Florence.gondret@inrae.fr

**Keywords:** broiler chickens, plant extracts, oxidative stability of meat, performance

## Abstract

**Simple Summary:**

The common practice of supplementing broiler feeds with oils rich in omega-3 fatty acids to improve the ratio of omega-6 to omega-3 fatty acids in chicken muscles results in a reduced oxidative stability of the meat and accelerated intensity of lipid peroxidation during storage. This study evaluated the effects of commercial rosemary (RO) and blackcurrant (BC) extracts rich in polyphenolic antioxidants added to broiler diets on the oxidative stability of meat and chicken production parameters. In this study, 120 one-day-old male Hubbard Flex broiler chicks were randomly allocated to 5 experimental groups, and for 35 days, were fed control starter and grower diets or the same basal diets containing two concentrations (2.5 and 5 g/kg) of the RO or BC extracts. The inclusion of RO and BC in the birds’ diet had no significant effect on broiler weight gain, feed conversion ratio, or carcass characteristics. The enrichment of chicken diet with RO and BC did not affect the oxidative stability of chicken breast muscles, but reduced the malondialdehyde (MDA) formation in frozen thigh muscles.

**Abstract:**

The effects of dietary supplementation with extracts of rosemary (RO) and blackcurrant (BC) on the performance indices and the oxidative stability of broiler meat were investigated during a 35-d experiment. For the experiment, 120 one-day-old male Hubbard Flex broiler chicks were randomly allocated to 5 experimental groups (control group and 4 treatments, each in 6 replications, 24 birds per group, 4 birds per replicate) and fed control starter and grower diets or basal diets containing two concentrations (2.5 and 5 g/kg) of the RO and BC extracts. Basic performance traits (body weight gain, feed intake, feed conversion) were recorded during the trial. At the end of the experiment, pectoral and thigh muscles were collected. Thiobarbituric acid (TBA) assays using the muscles samples were performed after 1 and 5 d of chilling (4 °C) and after 90 d of frozen storage (−18 °C). The inclusion of RO and BC in the birds’ diet had no significant effects on weight gain and feed conversion ratio of chickens, or on carcass characteristics, compared with control group. Enrichment of chicken diet with RO and BC did not affect the oxidative stability of chicken breast muscles, but the tested extracts significantly reduced the malondialdehyde (MDA) formation in frozen thigh muscles.

## 1. Introduction

Poultry meat is a rich source of protein, vitamins, and microelements, as well as n-6 and n-3 polyenoic fatty acids, which, at a specific ratio (4:1) in human diet, decrease the risk of coronary heart diseases by approximately 70% [1]. Broiler feed supplementation with oils rich in n-3 fatty acids (FA), e.g., linseed or rapeseed oil, is a common practice used for improving the ratio of n-6 to n-3 fatty acids in breast and leg chicken muscles, even to the level of 2.0–3.5:1 [2,3]. However, the increased content of polyenoic fatty acids in poultry meat resulted in a reduced oxidative stability of the material and accelerated the intensity of lipid peroxidation during storage. A “warmed-over” flavor is one of the negative effects of the muscle lipids oxidation and accumulation of the secondary derivatives. Some of these products, such as malondialdehyde (MDA) and oxysterols (oxyLDL) may have genotoxic effects [4] or may promote the development of cardiovascular diseases or cancer [5,6].

In the last decades, numerous studies were published on the utilization of rosemary and other herbs and spices, and plant by-products (incl. extracts, essential oils, leaves) in chicken nutrition. Rosemary (*Rosmarinus officinalis*) is a plant belonging to the Lamiaceae family, native to the Caucasus and to the Eastern Mediterranean, and introduced into many areas of the world [7]. Rosemary herb is commonly used as a food flavoring but is also known for its anti-inflammatory, antimicrobial, and antifungal activity [8,9]. The extracts of rosemary are suitable for use as food preservatives [10] because they have many polyphenolic compounds with antioxidant activity, and the extracts contribute to reducing lipid oxidation, inhibiting microbial growth, and extending the shelf-life of chicken broiler meat or other products during their storage [11]. The radical scavenging ability of methanol or acetone extract are comparable to commercial antioxidants, like α-tocopherol or butylated hydroxytoluene (BHT) [8].

The main active ingredients of rosemary include diterpenes (carnosic acid, 12-methylcarnosic acid, carnoside), cavotanoids (rosmarinic acid), and flavones (isoscutellarin-7-glucoside, genkwanin). Rosmarinic acid has the highest antioxidant activity and content in raw material [12]. The concentration of rosmarinic acid in rosemary leaves ranges from 0.2 to 4.3% [13].

Despite the effects of dietary rosemary byproducts on improving meat quality [14,15,16] and microbial balance in the gut of chickens [17,18], applications of potential polyphenols sources obtained from the byproducts of fruit processing for animal diets are still under investigation. Recent data included the enrichment of poultry diets with apple, blackcurrant, strawberry [19,20], and grape pomace [21] that have been shown to represent promising feed additives to improve tissue antioxidative traits and meat quality parameters. However, little is known about the use of highly purified polyphenolic extracts, obtained from fruit by-products, such as blackcurrant, used in juice processing [22].

Blackcurrant (BC; *Ribes nigrum*) is a plant species belonging to the Grossulariaceae family and cultivated in Europe and Northern and Central Asia. Poland is the primary producer and exporter of BC fruits, accounting for approximately 65% of world-wide production (120,000 tons per year) and exporting 80–90% of the total global exports [22]. BC fruits have high concentrations of polyphenolic compounds, anthocyanins, that cause their dark purple coloration. One gram of BC pomace contains approximately 65 mg of polyphenols [23]; however, commercial extracts are characterized by an average polyphenol concentration of 350–400 mg/g. The key anthocyanin components found in BC extracts include delphinidin-3-rutinoside, delphinidin-3-glucoside, peonidin-3-rutinoside, pelargonidin-3-rutoside, cyanidin-3-rutinoside and derivatives of myricetin. These substances are the main polyphenols found in BC preparations (160–300 g/kg). Other polyphenols including derivatives of quercetin, isoramnetin, or kaempferol [24].

The antioxidant activity of BC fruit compared to synthetic antioxidants is not thoroughly characterized in the available literature, but preliminary investigation of antioxidative activity indicated about 4.5-fold higher efficiency of BC extract in neutralization of the 2,2-diphenyl-1-picryl-hydrazyl free radical (DPPH), compared to butylated hydroxytoluene (BHT) [25]. Further investigations demonstrated that BC extract exhibited about 2–3 times higher antioxidant capacity than two analyzed extracts of rosemary. Supplemented diets of broiler chickens with identical concentrations of extracts characterized by the different antioxidant capacity will likely have a different effect on the inhibition of lipid peroxidation of chicken muscles. The present study, therefore, was aimed to evaluate the effects of commercial BC and RO extracts in broiler diets on the oxidative stability of meat and chicken production parameters. Both extracts were used in identical concentrations in the diets of chickens but exhibited diverse antioxidant capacity. The results of the experiment showed that further optimization of antioxidants addition to animal diets is needed.

## 2. Materials and Methods

### 2.1. Chemicals, Reagents, RO, and BC Extracts 

Commercial extracts of RO and BC were obtained from PK Components (Warsaw, Poland). Cyanidin-3-*O*-glucoside, a 3-*O*-rutinoside, delphinidin-3-*O*-glucoside, delphinidin-3-*O*-rutinoside, quercetin-3-*O*-glucoside, quercetin-3-*O*-rutinoside, myricetin-3-*O*-rutinoside, myricetin-3-*O*-galactoside, kaempferol-3-*O*-rutinoside, (+)-catechin, and (−)-epicatechin were purchased from Extrasynthese (Lyon, France). Neochlorogenic acid was obtained from TRANS MIT GmbH (Giessen, Germany). Acetonitrile was purchased from Merck (Darmstadt, Germany). The extracts were preliminarily tested for 1,1-diphenyl-2-picrylhydrazyl free radical (DPPH) to assess the synthetic capacity to neutralize the reactive oxygen species that are the main mediator of peroxidation process [25,26].

### 2.2. Identification and Quantification of Polyphenols by UPLC-PDA-MS Method

The qualitative (LC/MS Q-TOF) and quantitative (UPLC-PDA) analyses of polyphenols (rosmarinic acid, derivatives of gallocatechin, derivatives of luteolin, anthocyanins, flavan-3-ols, flavonols, and phenolic acids) were performed as described by Wojdyło et al. [27]. Polyphenols of the tested RO and BC extracts were identified using an ACQUITY Ultra Performance LC™ system with binary solvent manager, PDA detector (Waters Corporation, Milford, MA, USA) and a Micromass Q-TOF spectrometer (Waters, Manchester, UK) with an electrospray ionization (ESI) source operating in negative and positive mode. The separation of individual polyphenols was realized using a UPLC BEH C18 column (1.7 μm, 2.1 × 50 mm; Waters Corporation) at 30 °C. The results were expressed as mg per 1 kg of the extract of RO and BC.

### 2.3. Chemical Methods Used for the Diets and Extracts of RO and Blackcurrant 

The chemical composition of feed components was analyzed according to Association of Official Analytical Chemists [28] including: dry matter (DM): Zalmed SML 32/250 dryer (AOAC, 930.15); crude ash (CA) in muffle furnace (AOAC, 942.05); nitrogen by the Kjeldahl method (Sweden; AOAC, 984.13): Kjeltec 2300 Foss Tecator (Hillerod, Denmark); crude protein (CP) as 6.25 N, crude fat (CFA) by ether extraction: Büchi Extraction System B-811 (AOAC, 920.39); crude fiber (CF): Foss Fibertec 1020, Sweden (AOAC, 978.10).

### 2.4. Experimental Design and Diets

The study was carried out at the Wroclaw University of Environmental and Life Sciences experimental facilities (Research and Teaching Station in Swojczyce, Wrocław, Poland). In the 35-d trial, a total of 120 one-d-old Hubbard Flex broiler male chicks (37.3 ± 1.5 g) purchased from a local commercial hatchery were randomly divided into control and 4 experimental groups, each with 6 replications (battery cages with 4 birds per cage; 24 birds per group).

The ambient temperature was progressively reduced from initial 32 °C (1 d) to 22 °C (35 d), according to recommendations [29]. The lighting program included 18 h light and 6 h darkness. All procedures on animals were carried out with an approval of the Local Ethics Commission for Animal Experiments (Decision no. 67/2011) and were made in compliance with European Union and Ethical Commission regulations [30].

The composition of isoenergetic and isoprotein basal starter and grower diets given to the experimental chickens is shown in Table 1. The starter (d 1–14) and grower (d 15–35) diets were administered ad libitum in mash form. The basal starter control diet (I-C) contained 225 g/kg of crude protein (CP) and 12.30 MJ/kg metabolizable energy calculated according to World’s Poultry Science Association [31]. The CP and energy content in the grower control diet equaled 205 g/kg and 13.10 MJ/kg. Treatment starter and grower diets were based on the same basal control mixtures (I-C), supplemented with RO, group II RO 2.5 and III RO 5.0 g/kg) and BC extracts (group IV BC 2.5 g/kg and V BC 5.0 g/kg), applied in concentrations 2.5 g/kg (group II RO 2.5 and IV BC 2.5) and 5 g/kg (group III RO 5.0 and V BC 5.0). 

The basic chemical composition and polyphenolic fractions determined in the RO and BC extracts are presented in Table 2. The main component of the RO extract was rosmarinic acid that constituted 90.1% of total phenolic compounds. The phenolic acids were present as a sum of rosmarinic acid-3-*O*-glucoside and carnosic acid (7.5%). Additionally, rosemary extract gallocatechin derivatives (1.4%) and luteolin (1.0%) were quantified. The complete detailed polyphenolic composition found in BC extract was previously described in our associated publication [26]; therefore, the current composition is only presented as the main fractions. Briefly, the main polyphenolic fraction of BC consisted of anthocyanins (74.9%), followed by flavonols (14%), flavan-3-ols (6.1%), and derivatives of phenolic acids (4.9%). The BC extract (ethanolic extraction) contained 922.1 g/kg of dry matter and 18.1 g/kg of anthocyanins. The radical scavenging ability of the RO extract was about 2.4 times lower than the BC extract.

### 2.5. Experimental Measurements

The body weight (BW) of the chickens in each replicate (pen) was measured on day 1, 14, 29, and 35. The feed intake was recorded for day 1–14 (starter diet), day 15–28 (grower diet), and day 29–35 (total period of the experiment), and feed conversion indices (kg of feed intake per 1 kg BW gain) were calculated. On day 35, 2 randomly selected birds in each replicate were slaughtered, defeathered, and eviscerated. Breast muscles, liver, heart, gizzard, and abdominal fat were immediately excised from the carcass and weighed individually (all values were expressed as percentages of carcass weight). The pectoral and thigh muscles samples were collected to determine the development of lipid oxidation during storage at chilling (4 °C) and freezing conditions (−18 °C).

### 2.6. Determination of MDA Content in Fresh and Frozen Chicken Muscles

The extent of lipid peroxidation in breast and thigh muscles was determined by thiobarbituric (TBA) assay, after 1 and 5 d of storage under chilled conditions (4 °C) and after 90 d of frozen storage (−18 °C), according to the modified method of McDonald and Hultin [32]. The raw pectoral and thigh muscles were ground and homogenized. From the unified sample, 0.5 g of material was taken and 2 mL of 10% trichloroacetic acid (TCA) added, and the sample was homogenized, followed by centrifugation for 10 min at 4000× *g* (Sigma 3K30 Polygen). To 2 mL of the collected supernatant, 2 mL of 0.02 M TBA was added and stirred vigorously. The samples were then incubated at boiling water at temperature 99–100 °C (Julabo EcoTemp TW 12) for 40 min. After 20 min of cooling, the absorbance was read against the blank (TCA) at λ = 530 nm using Evolution 160 UV-VIS Thermo Scientific spectrophotometer. The results were calculated using the standard calibration curve based on the concentration of MDA pattern (1,1,3,3-tetramethoxypropane) from the absorbance and expressed as MDA content in mg per kg of muscle. The procedure was carried out in triplicate.

### 2.7. Statistical Analyses

All data obtained in this study were evaluated by one-way ANOVA and differences between the experimental factors were determined according to Tukeys’s Multiple Comparison Test using TIBCO Statistiva v. 13.3 Software [33]. The differences between treatments for all parameters were performed using the statistical model: yijk = µ + αi + eij, where yijk: variance associated with parameter; μ: overall mean; αi: the treatment effect; eij: the impact of specific factors.

## 3. Results

### 3.1. Diet Formulations and Performance Indices of Broiler Chickens

The effects of dietary supplementation of RO and BC extracts in different concentrations on average body weight gain and feed conversion, are summarized in Table 3.

From d 1 to 14 of age, no differences in body weight gain (BWG) of the chickens were observed. Feed conversion indices of chickens during application of the starter diet ranged from 1.45 kg/kg (I-C) to 1.60 kg/kg (V BC 5.0), with no differences among groups. During the second period of experiment (15–35 d), no differences in BW gains or feed conversion ratios were observed. No significant differences were noted also for the entire period of the trial. The average total BW gain of chickens could be presented as follows: 1778 g (I-C) > 1714 g (BC) > 1679 g (RO), with the feed conversion of 1.422 kg/kg BW gain (I-C) < 1.454 kg/kg BW gain (BC) < 1.506 kg/kg BW gain (RO). 

### 3.2. Carcass Characteristics

No differences (*p* > 0.05) in the relative weight of breast muscles, liver, gizzard, heart, edible offal, and abdominal fat were noticed (Table 4). The chicken carcass yields ranged from 74.60% (group III RO 5.0) to 76.48% (group V BC 5.0) and were significantly different from each other (*p* < 0.05). These differences among diets with extracts were not significant compared to the control group (I-C).

### 3.3. MDA Concentration

The effect of dietary supplementation with RO and BC extracts on lipid oxidation of chicken breast and thigh muscles stored at 4 °C for 1 and 5 d and in frozen state at −18 °C for 90 d is presented in Table 5. 

After 1 d of cold storage of the muscles, MDA content in pectoral muscles was not affected by diets supplemented with RO and BC extracts. The enrichment of broiler diets with 5.0 g/kg of RO (group III) and 2.5 g/kg of BC (group IV) showed significantly lower lipid oxidation in dark chicken meat in relation to control group (I-C). 

On d 5, MDA concentrations in breast chicken muscles were of 0.137 mg/kg (V BC 5.0) to 0.154 mg/kg (II RO 2.5), with no significant differences detected between the obtained values. In thigh muscles, significantly lower MDA formation was observed in samples obtained from chickens supplemented with 2.5 g/kg of BC (group IV), compared to muscles from groups I-C, II RO 2.5, and V BC 5.0. 

After 90 d of frozen storage, no significant impact of the antioxidant supplements on mg MDA per 1 kg of breast meat was noted. At the same time, thigh muscles from broilers fed diets enriched with RO and BC exhibited significantly lowered lipid oxidation expressed as MDA content (except for the group II RO 2.5), with the lowest MDA values recorded in thigh muscles sampled from group III RO 5.0 (0.210 mg/kg) and IV BC 2.5 (0.194 mg/kg). Clear effect of concentration-related diversification of MDA by tested additives was difficult to formulate; the RO 5.0 treatment showed to be more effective in protection of thigh muscles lipids against oxidation than the RO 2.5 (*p* < 0.05) and provided a similar degree of protection against peroxidation of frozen chicken dark meat as BC 2.5 treatment. In turn, increasing the concentration of BC to 5.0 g/kg of feed resulted in significantly higher MDA levels in thigh muscles in relation to BC 2.5 group (*p* < 0.01).

## 4. Discussion

### 4.1. Effects of the Polyphenolic Supplements on Bird Performance and Carcass Characteristics

The results of the present study showed no significant differences in performance between chickens fed diet containing various concentrations of RO and BC extracts or control diet. The available literature data showed some variations in performance of chickens fed diets containing RO by-products. Ghazalah and Ali [15] demonstrated that the 5–20 g/kg inclusion of RO leaves meal (RLM) to the grower feed (7–28 d) negatively affected the BW gain of birds, while a significant improvement in chicken performance was noted when administered the RLM to the finisher diet (29–49 d). Similar inconsistencies were published by Yesilbag et al. [16]. The authors observed significant increase of live weight gain and carcass yields in chickens fed diets containing RO volatile oils, but the performance traits and feed conversion ratio (FCR) were significantly affected after inclusion of RO leaves meal additives [16]. Significant decrease of body weight gain and feed intake but unaffected feed conversion ratio values were found after administration of 100 and 200 mg/kg of ethanolic extract of RO to chicken diets [34]. Higher caloric utilization in broilers fed diet with a 10 g/kg addition of RO leaves was also reported by Attia et al. [35]. In turn, Ghozlan et al. [36] confirmed no significant effect on broiler production after supplementation of diets with 5–15 g/kg dry RO leaves meal.

The reported variations in production performance of chickens fed different types of RO by-products may be due to the heterogeneous phenolics composition of the used supplements, as well as the type of raw material (and crude fiber content), method of administration, and concentrations of additives used in chicken diets. Lamaison et al. [13] determined the content of rosmarinic acid in the leaves of RO to be approximately 0.2 to 4.3%, and a similar variation of carnosic acid in 5 commercial RO extracts was found by Samotyja and Urbanowicz [37]. It has been shown by in vitro trials, that carnosic acid present in the RO extracts may inhibit pancreatic lipase, which decreased digestibility of fat and BWG in mice [38].

Chamorro et al. [39] claimed that an adverse effect of dietary polyphenols on performance traits might be observed with an excess proportion of these substances added to the diet (above 2.5 g/kg), which might have an adverse effect on ileal protein digestibility and negatively impact weight gain and feed conversion. However, our results do not confirm this hypothesis, because no negative effect on performance traits of chickens was observed even after addition of total phenolic compounds at the level of 4.40 g/kg of feed (III RO 5.0, Table 1). 

With the respect to BC extract, the main performance traits of broiler chickens were not affected by dietary BC inclusion, which agrees with our previous study [26] when the same BC extract was used. Similar findings have been reported in chickens fed 3 or 6% of apple, BC, strawberry [20], and chokeberry pomace [40].

In the experiment, no significant differences were noted in percentages of the weight of liver, heart, gizzard, and abdominal fat in carcass of chickens fed different levels of RO and BC compared to control group. These results were consistent with the data reported by other authors [20,41]. In turn, Ghazalah and Ali [15] noted a significant tendency to decrease the gizzard percentages as the level of RO leaves meal in the diet increased, but no significant effect of RML supplementation was found on other slaughter parameters. Oversized livers were reported in chickens receiving diet containing 7.5 g/kg of RO leaves [35], 25 g/kg of RO or nettle [40].

### 4.2. Effects of the Polyphenolic Supplements on Oxidative Stability of the Meat

The present study showed that inclusion of RO and BC extracts to the broiler diet can limit lipid oxidation in frozen thigh chicken meat, and thus can reduce the concentration of lipid oxidation derivatives. Similar findings have been reported when RO extract [14], essential oil of oregano [42] and grape seed pomace were used in poultry nutrition [43]. Loetscher et al. [40] also reported a strong inhibition of lipid peroxidation in breast muscles of broilers supplemented with RO leaves with a simultaneous elevation of tocopherol in meat. The inhibitory effect of dietary RO on the formation of MDA in chicken breast meat has also been reported [16,40]. 

The protective effect of RO and BC extracts against lipid peroxidation of the breast meat was, however, not found in our study. The lack of differences in TBA values in pectoral muscles of chickens fed diets enriched with 3 and 6% of apple, BC, or strawberry pomace were previously reported by Colombino et al. [20]. Also, Yong et al. [44] indicated that feeding broilers diet with grape powder significantly extended total phenolic content in the leg meat, with no differences detected in breast meat.

It should be emphasized that the biotransformation of polyphenols in the gut, as well as their absorption [45,46] and further deposition and metabolism in various tissues [47], may influence their biological activity in vivo. Other factors modulating the antioxidant activity of polyphenols include tissue-specific environment, where these substances could exert antioxidant or prooxidant properties, or/and endogenous signaling mechanisms, including regulation of gene expression [48]. In thigh muscles peroxidation proceeds more intensively, which results primarily from the higher lipid and endogenous pro-oxidants content (i.e., transition metals and haem-proteins) [49] participating in the Fenton reaction, providing a source of reactive oxygen species.

When compared with RO, the BC extract used at concentration of 2.5 g/kg was more effective in reducing the lipid oxidation of cold stored (5 d) and frozen (90 d) thigh muscles and exerted protective effect against peroxidation of frozen dark meat as the dietary RO at the level of 5.0 g/kg. Flavanol fractions present in BC extract exert strong antioxidant capacity because their substitution with 3′,4′-dihydroxy in the B-ring and conjugation between the A- and B-rings. Also, flavones, in general, exhibit higher antioxidant activity compared to anthocyanins with the same hydroxylation patterns measured with the ORAC assay [50]. The 2–3 times higher activity of the BC extract in relation to RO was also confirmed towards DDPH free radical assay [26].

The protective effect of BC extract towards lipoperoxidation of frozen thigh meat was, however, significantly lower when compared to BC 2.5 treatment, and was not confirmed in dark meat samples stored for 5 days. In our previous study [26], the inhibiting effect of BC extract towards lipid peroxidation of thigh muscle was revealed only during long-term storage (90 d, −18 °C). Moreover, a significant pro-oxidative effect has been observed in pectoral muscle which related to the potentially pro-oxidative properties of the selected polyphenolic compounds identified in the BC extract [26]. No other comparable studies on the effect of BC extract supplementation were found on chicken meat susceptibility to oxidation processes. However, the obtained effect and differences between the two experiments including the application of BC extract in broilers nutrition may be linked to both the different concentration (previously 1.125 g and 2.5 g of BC/kg of feed) of BC and the duration of the supplementation period (last 10 or 20 days of the previous experiment).

Another potential explanation for the obtained differences may be related to intracellular mechanisms involved in the regulation of oxidative stress. The main regulator of this process is the nuclear factor-erythroid-2-related factor 2 (Nrf2) that controls the expression of antioxidant genes in response to reactive oxygen species (ROS) production. Marked, but not concentration-dependent, increase in expression of intracellular catalase and the Nrf2 was found in mammary cell cultures, incubated with various concentrations of baicalin (Scutellaria baicalensis) [51]. The addition of baicalin had a strong, but not linear antioxidant effect on the adipose and muscle tissue of pigs [52]. The acute exposure (90 min) of baicalin to mammary culture cells caused a more visible decrease in the amount of ROS production compared to chronic exposure (24 h); moreover, the inhibitory effect of baicalin towards ROS formation was lower at high concentrations (i.e., 100 or 200 µg/mL) than at lower concentration (1–50 µg/mL) [50]. Although direct comparison of the cited in vitro data to our in vivo results cannot constitute confirmation of our insights, it may illustrate an interesting mechanism that may occur in various tissues under the influence of selected sources of antioxidants. 

## 5. Conclusions

The results demonstrate than enrichment of broilers diets with RO and BC extracts did not significantly affect broilers’ weight gain, feed conversion ratio, as well as the carcass characteristics. The inclusion of dietary RO and BC extracts did not significantly affect the oxidative stability of chicken breast muscles, while broiler diets supplemented with RO and BC increased the oxidative stability of thigh muscles during frozen storage and tended to decrease the MDA concentration in thigh muscles during storage in chilled conditions. At the same time, the BC extract proved to be a more potent source of antioxidants and at level of 2.5 g/kg of feed showed a higher inhibitory potential in lipid peroxidation processes in dark chicken meat compared to the RO extract. However, a further clarification of potential intracellular antioxidative mechanisms in various conditions is required to predict the possible side effects of natural antioxidants applied in animal nutrition.

## Figures and Tables

**Table 1:** Composition of the experimental starter and grower diets fed to broilers.

Diets Groups	Starter [g/kg]					Grower [g/kg]				
	I-C Control	II RO 2.5	III RO 5.0	IV BC 2.5	V BC 5.0	I-C Control	II RO 2.5	III RO 5.0	IV BC 2.5	V BC 5.0
Maize	250.00	247.5	245.00	247.5	245.00	250.00	247.5	245.00	247.5	245.00
Wheat	261.00	261.00	261.00	261.00	261.00	286.10	286.10	286.10	286.10	286.10
Soybean meal	403.20	403.20	403.20	403.20	403.20	364.80	364.80	364.80	364.80	364.80
Rapeseed oil	43.60	43.60	43.60	43.60	43.60	59.10	59.10	59.10	59.10	59.10
Chalk	10.30	10.30	10.30	10.30	10.30	12.60	12.60	12.60	12.60	12.60
Dicalcium phosphate	18.90	18.90	18.90	18.90	18.90	14.30	14.30	14.30	14.30	14.30
Sodium chloride	3.00	3.00	3.00	3.00	3.00	3.10	3.10	3.10	3.10	3.10
Vitamin/mineral premix *	10.00	10.00	10.00	10.00	10.00	10.00	10.00	10.00	10.00	10.00
RO or BC extract **	0.00	2.50	5.00	2.50	5.00	0.00	2.50	5.00	2.50	5.00
Energy Value [MJ] and Essential Nutrients [g/kg] of Experimental Diets										
ME [MJ/kg]	12.30	12.27	12.23	12.27	12.23	13.10	13.06	13.03	13.06	13.03
Dry matter [g/kg]	896.20	896.28	896.36	896.26	896.33	898.70	898.77	898.84	898.76	898.82
Crude protein [g/kg]	225.0	224.46	223.92	224.44	223.88	205.00	204.51	204.02	204.49	203.98
Crude fiber [g/kg]	33.20	33.12	33.04	33.33	33.45	32.00	31.92	31.85	32.13	32.26
Crude fat [g/kg]	62.10	61.96	61.81	62.02	61.93	88.10	87.89	87.68	87.95	87.80
Crude ash [g/kg]	58.30	58.21	58.13	58.18	58.06	55.60	55.52	55.44	55.49	55.37
Nitrogen free extractives [g/kg]	517.60	518.53	519.46	518.30	519.01	518.00	518.93	519.85	518.70	519.41
Ca [g/kg]	10.30	10.29	10.28	10.29	10.28	10.30	10.29	10.28	10.29	10.28
P available [g/kg]	5.00	4.99	4.99	4.99	4.99	4.70	4.69	4.69	4.69	4.69
Na [g/kg]	1.70	1.69	1.69	1.69	1.69	1.70	1.70	1.69	1.70	1.69
Concentration of Active Substances in RO and BC Preparations Added to the Diets [g/kg]										
Rosmarinic acid [g/kg]	0.00	1.98	3.96	0.00	0.00	0.00	1.98	3.96	0.00	0.00
Anthocyanins [g/kg]	0.00	0.00	0.00	0.045	0.090	0.00	0.00	0.00	0.045	0.090
Total of phenolic compounds [g/kg]	0.00	2.20	4.40	0.60	0.120	0.00	2.20	4.40	0.60	0.120

* Chemical composition of the premix per 1 kg of diet: CaCO_3_—0.9 g, P—0.8 g, S—250 µg, Mn—80 mg, J—1 mg, Zn—80 mg, Fe—70 mg, Co—400 µg, Se—300 µg, retinol—7.8 mg, cholecalciferol—75 µg, α-tocopherol—60 mg, menadione—3.25 mg, thiamin—3.03 mg, riboflavin—8 mg, pyridoxine hydrochloride—5.53 mg, cyanocobalamin—30 µg, pantothenic acid—15 mg, biotin—200 µg, nicotinic acid—60 mg, folic acid—2 mg, choline—500 mg, Lys—1.85 g, Met—2,25 g, Tre—0.6 g, phytase, coccidiostat—salinomycin, prebiotic. ** Amount of supplemented extract of RO or BC added to 1 kg of the mixture.

**Table 2:** Basic chemical composition and main polyphenolic fractions determined in the rosemary and blackcurrant extracts.

Tested Extract	Extract of Rosemary (RO)		Extract of Blackcurrant (BC) [26] *		
Compounds	Content in [g/kg]	Content in [%]	Content in [g/kg]	Content in [g/kg]	Content in [%]
Rosmarinic acid	792.0	90.1	Anthocyanins	1.81	74.9
Derivatives of phenolic acids	66.21	7.5	Derivatives of phenolic acids	0.12	4.9
Gallocatechin derivatives	12.11	1.4	Flavonols	0.34	14.1
Derivatives of luteolin	8.74	1.0	Flavan-3-ols	0.15	6.1
Total	879.06	100.0	Total	2.42	100.0
Basic Composition [g/kg]					
Dry matter	927.40		922.1		
Crude ash	23.4		1		
N × 6.25	9.7		83.6		
Crude fiber	1		28.3		
Ether extract	4.6		10.2		
Nitrogen free extractives	888.7		799.0		
Radical Scavenging Ability (mean ± SD) [26] *					
IC_50_^DPPH^ [mg/L] **	26.36 ± 0.85		10.68 ± 0.83		

* The preliminary data cited in our previous study. ** Half maximal inhibitory concentration of the tested extracts toward DPPH free radical.

**Table 3:** Average body weight and feed conversion of broiler chickens.

	Weight Gain [g]			Feed Conversion [kg feed/kg BW Gain]		
	D 1–14 (Starter)	D 14–35 (Grower)	D 1–35 (Total)	D 1–14 (Starter)	D 14–35 (Grower)	D 1–35 (Total)
Group						
I-C (control feed)	295	1483	1778	1.45	1.42	1.42
II RO 2.5 (2.5 g of RO/kg of feed)	298	1389	1687	1.50	1.51	1.51
III RO 5.0 (5.0 g of RO/kg of feed)	294	1377	1671	1.57	1.50	1.51
IV BC 2.5 (2.5 g of BC/kg of feed)	282	1484	1766	1.57	1.40	1.43
V BC 5.0 (5.0 g of BC/kg of feed)	286	1377	1663	1.60	1.45	1.48
SEM	4.403	21.977	22.564	0.025	0.032	0.026
*p* value (group)	0.787	0.272	0.335	0.316	0.803	0.761

I-C—control group, RO—chicken fed rosemary extract, BC—chicken fed blackcurrant extract.

**Table 4:** Effects of dietary treatments on slaughter yields and proportions of selected carcass organs.

	Breast Muscles [%]	Liver [%]	Stomach [%]	Heart [%]	Edible Offal [%]	Abdominal Fat [%]	Slaughter Yield [%]
Group							
I-C (control feed)	26.34	2.59	1.49	0.70	4.78	0.62	74.82 ^ab^
II RO 2.5 (2.5 g of RO/kg of feed)	26.03	2.61	1.41	0.74	4.75	0.54	74.86 ^ab^
III RO 5.0 (5.0 g of RO/kg of feed)	25.66	2.47	1.44	0.72	4.63	0.64	74.60 ^a^
IV BC 2.5 (2.5 g of BC/kg of feed)	25.20	2.56	1.43	0.79	4.77	0.63	75.80 ^ab^
V BC 5.0 (5.0 g of BC/kg of feed)	25.32	2.40	1.39	0.72	4.51	0.73	76.48 ^b^
SEM	0.327	0.046	0.03	0.017	0.058	0.043	0.222
*p* value (group)	0.803	0.575	0.889	0.576	0.548	0.732	0.029

I-C—control group, RO—chicken fed rosemary extract, BC—chicken fed blackcurrant extract. ^a,b^ Means in the column with different superscripts differ significantly at *p* < 0.05.

**Table 5:** TBA values, presented as MDA content [mg/kg] in breast and thigh muscles stored at 2–3 °C for 1 and 5 d, or stored at −18 °C for 90 d.

	Days of Storage					
	1	5	90	1	5	90
	Pectoral Muscles			Thigh Muscles		
Group						
I-C (control feed)	0.118	0.141	0.199	0.174 ^Aa^	0.210 ^A^	0.249 ^A^
II RO 2.5 (2.5 g of RO/kg of feed)	0.138	0.154	0.213	0.144 ^ABab^	0.207 ^A^	0.235 ^ABa^
III RO 5.0 (5.0 g of RO/kg of feed)	0.118	0.143	0.201	0.137 ^b^	0.195 ^AB^	0.210 ^BCb^
IV BC 2.5 (2.5 g of BC/kg of feed)	0.110	0.148	0.198	0.125 ^B^	0.182 ^B^	0.194 ^C^
V BC 5.0 (5.0 g of BC/kg of feed)	0.116	0.137	0.200	0.155 ^ABab^	0.201 ^A^	0.221 ^B^
SEM	0.004	0.003	0.003	0.004	0.002	0.004
*p*-value (group)	0.233	0.337	0.502	0.002	0.000	0.000

I-C—control group, RO—chicken fed rosemary extract, BC—chicken fed blackcurrant extract. ^a,b^ Means in the column with different superscripts differ significantly at *p* < 0.05. ^A,B,C^ Means in the column with different superscripts differ significantly at *p* < 0.01.

## Data Availability

The data [radical scavenging ability of the rosemary and blackcurrant extract, performance indices of broiler chickens, MDA concentration] used to support the findings of this study have been deposited in the “Additives of natural polyphenolic extracts to the broiler chickens diet and their effect on oxidative stability of meat and selected production parameters” repository, Ph.D. thesis (in Polish): fbc.pionier.net.pl/details/oai:www.dbc.wroc.pl:23332 (accessed on 16 April 2021). The quantification of polyphenols of rosemary and blackcurrant extract data used to support the findings of this study are available from the corresponding author upon request.
